# The Implementation of HIV Self-Testing in Resource-Limited Settings Where the HIV Disease Burden is High

**DOI:** 10.3389/ijph.2023.1605790

**Published:** 2023-05-17

**Authors:** Pachamuthu Balakrishnan, A. S. Smiline Girija, Saravanan Shanmugam, Iyanar Kannan, Ramachandran Vignesh, Esaki M. Shankar, Sree T. Sucharitha

**Affiliations:** ^1^ Department of Microbiology, Centre for Infectious Diseases, Saveetha Dental College and Hospitals, Saveetha Institute of Medical and Technical Sciences (SIMATS), Chennai, India; ^2^ Global Biomedical Research Foundation, Chennai, India; ^3^ Preclinical Department, Faculty of Medicine, Royal College of Medicine Perak, Universiti Kuala Lumpur, Ipoh, Malaysia; ^4^ Infection and Inflammation, Department of Biotechnology, Central University of Tamil Nadu, Thiruvarur, India; ^5^ Department of Community Medicine, Annaii Medical College and Hospital, Sriperumbudur, Tamil Nadu, India

**Keywords:** WHO, HIV/AIDS, 95-95-95 target, HIV self-testing, policy making, resource-limited settings, high-risk population, first-time tester

## Abstract

In resource-limited settings, there is growing evidence that HIV testing is lacking among high-risk key populations such as men having sex with men, injection drug users, and transgenders largely due to stigma, discrimination, and lack of confidentiality. Findings from recent studies among high-risk key populations and the general population from various regions including resource-limited settings support the need for wider accessibility of HIV self-testing (HIV-ST) to reach those who may not otherwise have access to testing. Therefore, HIV-ST has untapped potential as a strategy to improve access to HIV testing and to increase testing frequency among key high-risk populations and their partners. Though HIV-ST has emerged as a safe, acceptable, and effective way to reach people, there are several roadblocks to implementing the HIV-ST policy, and fast-track policy implementation needs to be necessitated with newer or modified strategic plans.

## Background

Having claimed >40 million lives thus far, HIV continues to remain a major global public health burden. Recent World Health Organization (WHO) estimates suggest that there were ∼38.4 million people living with HIV (PLHIV) at the end of 2021 [1]. The Joint United Nations Programme on HIV/AIDS (UNAIDS) has set a target for achieving “*95-95-95*” by 2030 where the target is to diagnose 95% of all HIV-positive individuals, provide antiretroviral therapy (ART) for 95% of those who are diagnosed, and achieve viral suppression among 95% of the treated subjects [2]. Nonetheless and despite the blitzkrieg efforts to prevent and control HIV, only 76% of PLHIV are aware of their infection status as per available data [3].

The WHO introduced the HIV self-testing (HIV-ST) guidelines [4] in 2016 with a strong recommendation wherein a person is able to collect his or her own biological specimens (such as blood and saliva), perform the self-test, and can self-interpret the results to be aware of his/her HIV status. There are several WHO pre-qualified HIV-ST kits available in the market such as Wondfo HIV Self-Test (Wondfo Biotech Co., Ltd., Guangzhou, China), CheckNOW HIV Self-Test (Abbott Rapid Diagnostics, Jena, Germany), SURE CHECK HIV Self-Test (Chembio Diagnostic Systems, Inc., Medford, USA), Mylan HIV Self-Test (Atomo Diagnostics Pvt. Ltd., Leichardt, Australia), INSTI HIV Self-Test (bioLytical Laboratories Inc., Richmond, Canada), and OraQuick HIV Self-Test (OraSure Technologies, Inc., Bethlehem, USA). Importantly, HIV self-screening is viewed by the WHO as a triage strategy that stands to complement facility-based testing for scaling up the numbers to achieve the UNAIDS target. These screening tests will encourage accessibility to HIV testing across all walks of the global population. Further, recently available evidence also suggests that HIV self-testing when directed toward individuals at risk of contracting the infection would be cost-effective and largely helpful in early and prompt diagnosis of HIV infection [5, 6]. Hence, the current concept article is set to shed some light on the challenges and opportunities in our way ahead of effectively implementing HIV-ST in resource-limited settings based on the WHO guidelines and evidence-based research reports.

### Current Status of HIV-ST Implementation

As HIV-ST is a convenient, confidential, and enabling way to achieve the target goals, HIV self-tests are largely available in the United States and many countries across Europe and are increasingly becoming accessible throughout sub-Saharan Africa and other parts of the world including the Asia-Pacific that have included HIV-ST as a national policy or strategic plan [7]. In 2020, the WHO reported that 88 countries worldwide had already introduced HIV-ST and 31 other countries were developing policies and strategic plans to be implemented [8]. However, about two-thirds of these nations including Australia, Brazil, France, Moldova, the UK, and the USA have upper-middle or high-income status. In 2019, the first HIV-ST kit that uses urine specimens was approved by the Chinese Food and Drug Administration (CFDA), which largely promoted the use of HIV self-testing across China [9]. Moreover, several other countries across the world including India are currently engaged at different levels of policy decisions and implementations but at a slow pace.

### Challenges Faced in Implementing HIV-ST

One of the major concerns about HIV-ST is the ability for incorporating pre- and post-test counseling and linkage to confirmatory testing and clinical care. Though several methods have been employed to incorporate pre- and post-test counseling with self-testing via test kit pack inserts, toll-free hotline numbers, mobile applications, text messages, websites, pre-recorded online videos, and online video counseling [10–13], the expected effectiveness of these methods largely remain questionable to generalize the concepts globally though there are some limited data available. Other key challenges are the cost of the HIV-ST kit, the potential for inaccurate results, difficulty ensuring linkage to clinical care, self-testing as a justification for unprotected sex, and unsafe disposal of biohazard material after use. Importantly, the cost factor is highly critical for low- and middle-income countries (LMICs). However, the cost of HIV-ST has been reduced to 2 US$ (ex-works) for the LIMCs with the buy-down support by the Bill and Melinda Gates Foundation, USA, and this is applicable to HIV-burdened countries such as South Africa, Nigeria, and India as per the WHO/UNITAID Report 2020.

### Critical Analysis

Globally, apart from pre- and post-test counseling and linkage to clinical care, there have been miscellaneous concerns about HIV-ST, which vary from test accuracy, test misuse, illiteracy of the people where the HIV burden is high, and awareness among the users on the availability and accessibility to the kits before implementing the policy in resource-limited settings. Though there are some sporadic reports available to address these areas, there remains a paucity of data for large-scale implementation [14, 15]. Moreover, these factors do not encourage policymakers to implement HIV-ST instantaneously, entailing a substantial delay (or hesitancy) for policy enactment in a vast majority of the resource-limited settings where HIV-ST remains to be implemented.

#### Scale-Up Approach for HIV-ST

**FIGURE 1:**
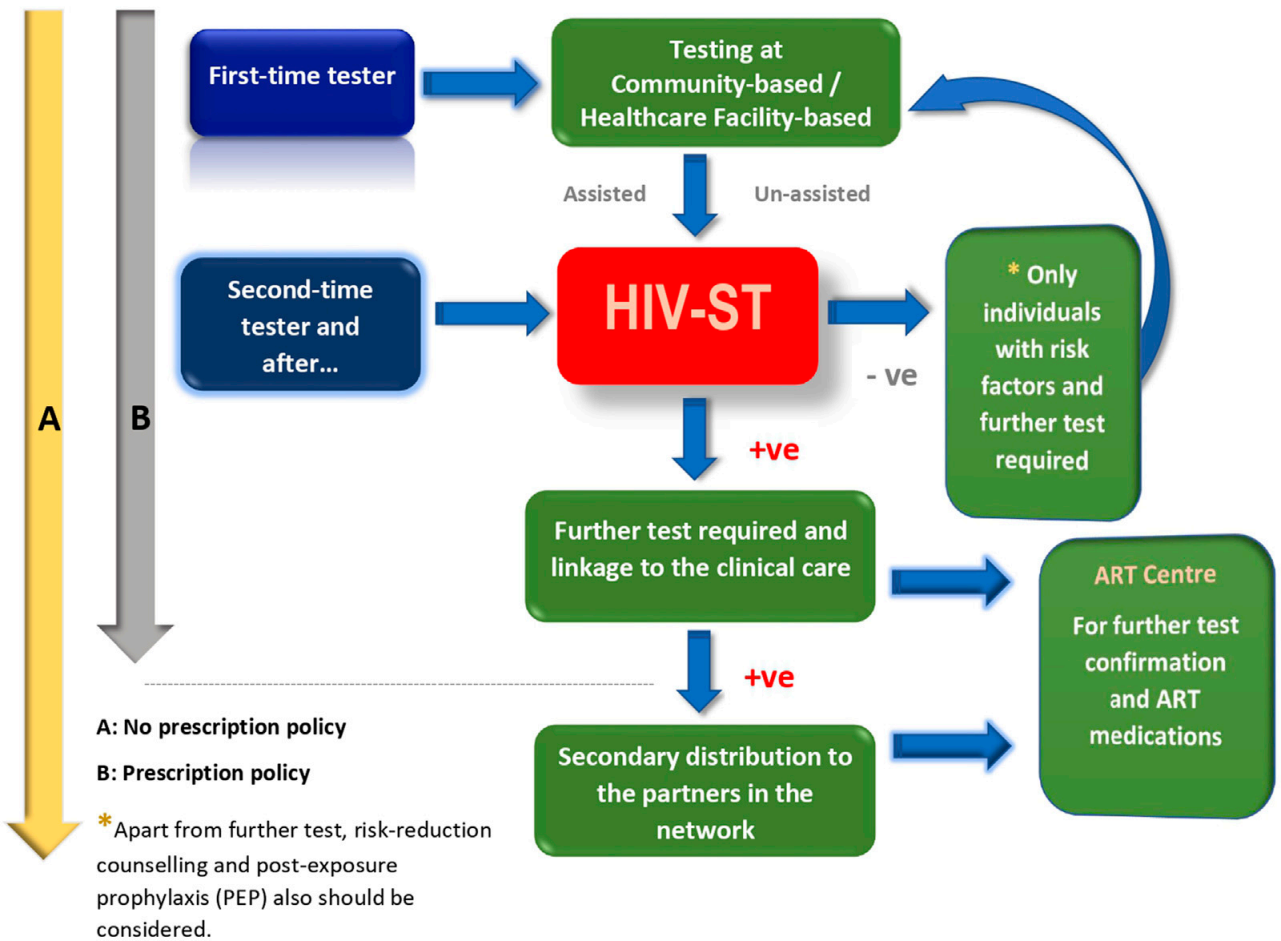
Safer scale-up of HIV-ST for the high-risk key populations (e.g., MSM and IDU) with and without prescription policies. (India 2023).

The data that are available from population studies conducted across high-income and resource-limited countries with diverse self-testing approaches (assisted and un-assisted) clearly indicate a very high level of acceptability for HIV self-testing [10]. The HIV-ST has the advantage of providing a greater level of privacy in contexts where HIV-related stigma is highly prevalent [16, 17], and, hence, the HIV-ST would offer an innovative approach to scale up and encourage testing by people who are reluctant to get tested in formal healthcare settings and an effective way to reach people who may not test otherwise [18]. The HIV-ST approach not only bears the potential to increase or scale up accessibility to HIV testing across the different population groups, but it would also prove to be helpful in the early diagnosis, besides being cost-effective, for use among the high-risk groups [5, 6, 19]. Studies also suggest that the secondary distribution of HIV-ST kits via peers, sexual partners, and female sex workers (FSWs) has largely remained successful and appears to be convenient, ensures a high degree of confidentiality, and also allows couples to test privately [20–22] and have a linkage to clinical care [23]. We propose the scale-up model for HIV-ST in high-risk populations which could potentially enhance the testing and detection of more HIV-infected individuals (Figure 1). After first-time testing with appropriate counseling procedures, the client will have sufficient confidence to handle HIV-ST without much anxiety. Though the proposed model is for high-risk populations with or without a prescription policy, this approach could also be potentially implemented for the general population as well.

Recently, WHO conducted a systematic review [4] to update the guidance on HIV-ST based on 32 randomized-controlled trials (RCTs) compared with standard facility-based HIV testing. The key messages from the RCTs were: a) HIV-ST increases the adoption of HIV testing and a majority of the people are willing to and can perform HIV-ST with minimal support, b) Misuse of HIV-ST and social harms associated with HIV-ST are apparently rare and, importantly, no suicides have been reported, c) Proportions of people diagnosed and linked to care with HIV-ST are comparable to those with standard facility-based testing, and d) HIV-ST does not increase sexual risk behavior among men who have sex with men (MSM), which represents one of the key groups of the global population.

The study by Empel et al. [11] from the sub-Saharan African (SSA) region with the aim of understanding the patterns of HIV-ST awareness and utilization in nine SSA countries revealed that rural, less educated, and lower-income populations were least likely to have heard of or used HIV-ST [11] and documented that ∼98% of the study participants had never self-tested. On the other hand, the consistent increase in self-testing across the higher socio-economic and well-educated groups appears to correspond to increased HIV-ST usage. Importantly, the lower socio-economic groups have been shown to possess a higher risk of acquiring HIV [24] and a similar scenario has also been documented in Thailand, recently [25]. It has also become evident that a successful implementation of HIV-ST across Africa has resulted in a high HIV positivity rate among individuals who previously appear to have remained undiagnosed [15,16].

As per the WHO, HIV-ST is a safe and effective way to reach out to people who are at high risk and has been recommended as a convenient and confidential modality for HIV testing. Nonetheless, all reactive HIV-ST results must be referred to further testing by a healthcare provider to confirm HIV status as per the national testing algorithm. Further, retesting following a negative self-test result is necessary only for those who are at active risk such as people from key populations (MSM, IDU, and sex workers) and those who report potential HIV exposure in the preceding 12 weeks [4]. However, false positive test results do exist with multiple HIV testing platforms, which may result in devastating consequences [26–29].

India represents the third largest country in terms of HIV disease burden, and studies on HIV oral self-screening among HIV/STD/TB clinic attendees have shown that there is a high level of concordance in HIV-ST results interpreted by participants and physicians [30]. The higher rates of acceptance for HIV-ST have also been documented among truck drivers, FSWs, MSM, transgenders (TG), youths, and peer educators [31–33]. A study supported by the National AIDS Control Organization (NACO), Government of India, New Delhi, has revealed that awareness about HIV-ST is inadequate but HIV-ST is highly acceptable to the key populations. Interestingly, MSMs preferred un-assisted HIV-ST more than the other key populations [14]. The key populations also identified challenges such as the absence of pre- and post-HIV test counseling, linkage to clinical care, cost, and potential coercion as barriers to HIV-ST.

#### Blood Versus Non-Blood for HIV-ST

A global literature review has revealed [10, 32] that key populations preferred the oral fluid-based HIV-ST over the blood-based test as oral fluid-based tests are more convenient and easier to perform, but both the sensitivity and specificity of the oral fluid-based tests are relatively lesser than blood-based HIV-ST. Moreover, it is important to note that the users are likely to make errors when blood-based self-tests are performed due to “*fear of needle prick*” and the tests become invalid [18].

The use of urine specimens for HIV-ST has grossly been neglected in the past and the probable rationale appears to be the poor sensitivity as compared with oral-fluid or blood with the earlier urine-based test kits. However, the currently available HIV-ST urine-based test kit has been shown to be highly effective in diagnosing HIV infection. The performance of the HIV-ST kit (approved by the CFDA) with urine was evaluated in a multi-centric large-scale cohort in China. The HIV antibody detection concordance of colloidal gold test kit with urine and blood was 98.07% among HIV-positive individuals and 100% in healthy individuals [9]. The antibody detection concordance between un-assisted self-testing with urine by individuals and professionals was 99%. Recently, as a novel approach, the effectiveness of anonymous vending machine-based urine self-collection for HIV testing programs was assessed among college students across China and was shown to be a powerful tool to promote HIV testing [34]. The US Food and Drug Administration (US-FDA) licensed an enzyme immunoassay for the detection of HIV-1 antibodies in urine in 2019 [35] and advocates for urine in the screening of high-risk populations as the reports show that screening urine could serve as an improved alternative to oral specimens [36]. Akin to oral fluid-based HIV-ST, urine can also be disposed of safely after use.

### Conclusion

HIV diagnosis remains a necessary precursor to ART and viral suppression, which are the other two targets of the “*95-95-95*” and can prevent disease transmission substantially. Greater and continuous awareness about the possibility of false positives and negatives with screening tests among key high-risk populations and the general population should be in place where HIV-ST policy is implemented to translate to improved uptake with good levels of understanding. A growing evidence base underscores the feasibility, reliability, safety, and acceptability of HIV-ST across the population, and scaling up HIV-ST needs to be urgently promoted with due policy decisions. Furthermore, fast-track policy implementation needs to be necessitated with newer or modified strategic plans.
